# Helminth Sensing at the Intestinal Epithelial Barrier—A Taste of Things to Come

**DOI:** 10.3389/fimmu.2020.01489

**Published:** 2020-07-30

**Authors:** Aduragbemi A. Faniyi, Kevin J. Wijanarko, James Tollitt, John J. Worthington

**Affiliations:** ^1^Biomedical and Life Sciences, Faculty of Health and Medicine, Lancaster University, Lancaster, United Kingdom; ^2^Institute of Inflammation and Ageing, University of Birmingham, Birmingham, United Kingdom; ^3^Department of Biomedical Sciences, City University of Hong Kong, Kowloon, Hong Kong

**Keywords:** tuft cells, enteroendocrine cell (EEC), microbiome, epithelium, helminth, G protein-coupled receptor (GPCR)

## Abstract

Human intestinal helminth infection affects more than 1 billion people often in the world's most deprived communities. These parasites are one of the most prevalent neglected tropical diseases worldwide bringing huge morbidities to the host population. Effective treatments and vaccines for helminths are currently limited, and therefore, it is essential to understand the molecular sensors that the intestinal epithelium utilizes in detecting helminths and how the responding factors produced act as modulators of immunity. Defining the cellular and molecular mechanisms that enable helminth detection and expulsion will be critical in identifying potential therapeutic targets to alleviate disease. However, despite decades of research, we have only recently been able to identify the tuft cell as a key helminth sensor at the epithelial barrier. In this review, we will highlight the key intestinal epithelial chemosensory roles associated with the detection of intestinal helminths, summarizing the recent advances in tuft cell initiation of protective type 2 immunity. We will discuss other potential sensory roles of epithelial subsets and introduce enteroendocrine cells as potential key sensors of the microbial alterations that a helminth infection produces, which, given their direct communication to the nervous system *via* the recently described neuropod, have the potential to transfer the epithelial immune interface systemically.

## Introduction

Soil-transmitted helminths (STHs) affect >1 billion people in the world's most deprived communities (1). These parasites are one of the most prevalent neglected tropical diseases worldwide bringing huge morbidities to the host population. Sub-Saharan Africa alone is estimated to lose 2.3 million disability-adjusted life-years annually (2). Constant advances have been made in identifying type 2 immune responses as key to helminth control and expulsion (3–8), with the cytokine interleukin (IL)-13 being crucial in driving the characteristic “allergic” immune response (3). CD4+ T-cells and more recently type 2 innate lymphoid cells (ILC2s) are key producers of these cytokines, with ILC2 believed to be the major initiators of type 2 immunity (9, 10) driven by the release of the epithelial alarmins IL-33, thymic stromal lymphopoietin (TSLP), and IL-25 (11).

**Figure 1:**
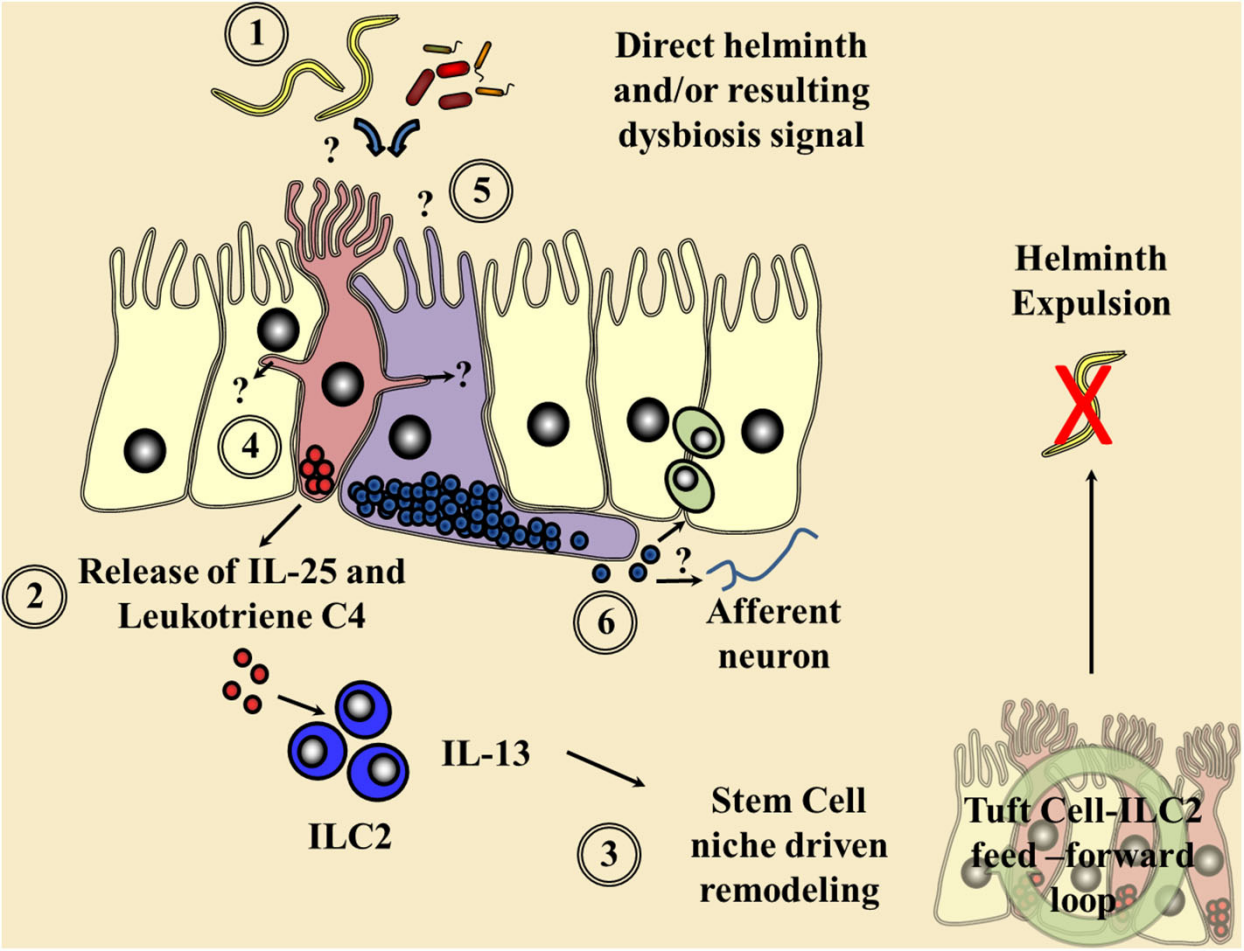
Current understanding of chemosensory detection of helminths at the epithelial barrier and a flavor of possible future perspectives. (1) Helminths are detected by tuft cells (red) through an as yet undefined receptor and ligand, although microbial dysbiosis produced *via* helminth colonization may be a potential candidate. (2) Gustducin-α and the transient receptor potential cation channel, subfamily M, member 5 (TRMP5) are required for the signaling cascade and Ca2+ flux, allowing the secretion of the alarmin interleukin (IL)-25 and leukotriene C4 in an arachidonate 5-lipoxygenase (ALOX5)-dependent mechanism signaling to resident type 2 innate lymphoid cells (ILC2s). (3) These factors in turn increase ILC2 numbers and their secretion of the cytokine IL-13, driving a feed-forward loop *via* the stem cell niche resulting in helminth expulsion. Tuft cell-derived acetylcholine could also possibly alter this epithelial stem cell niche and local immune responses. (4) Potential cross communication of tuft cells *via* cytospinules and the relay of helminth-derived signals to coordinate surrounding epithelial response. (5) The potential of enteroendocrine cells (purple), which host an array of chemosensory apparatus, to directly sense a helminth infection or infection-induced microbial dysbiosis. (6) The release of enteroendocrine peptide hormones signaling to the surrounding immune system either directly or *via* neuronal communication is proposed.

Following the identification that tuft cells of the rat epithelium (12) possessed alpha-gustducin, the G-protein subunit of numerous taste receptors, it had been postulated that these cells could act as solitary chemosensory apparatus within tissues. The identification of the tuft cell-specific marker doublecortin like kinase 1 (DCLK1) (13–17) and the discovery of the downstream master transcription factors, Pou domain, class 2, transcription factor 3 (*Pou2f3*) and growth factor-independent 1b (*GFI1b*) (15, 18–20), allowed further elucidation of this tuft cell chemosensory hypothesis. In 2016, three key papers cemented the importance of tuft cells in sensing parasites and brought tuft cell biology to the forefront of helminth immunity (21). Through examination of Pou2f3 null mice during a small intestinal helminth infection, Gerbe et al. (19) defined that IL-13 acted downstream of the tuft cell lineage, suggesting a tuft cell initiated IL-25-driven positive feed-forward loop resulting in ILC2 expansion and IL-13-driven tuft and goblet cell hyperplasia (Figure 1), essential for helminth expulsion (22, 23). In parallel to these studies, von Moltke et al. (24) confirmed tuft cells as IL-25 expressers that, following small intestinal helminthiasis, underwent hyperplasia *via* an ILC2 derived IL-13 interaction with the IL-4Rα in a feed-forward loop, presumably *via* stem cell niche signaling (25, 26). Finally, mice null for the G-protein subunit gustducin or the transient receptor potential cation channel, subfamily M, member 5 (TRMP5), a cation channel known to be important in the signaling cascade of chemosensory cells in the gut, mirrored the delayed tuft and goblet cell hyperplasia following a small intestinal helminth infection, giving the first indication of the chemosensory mechanisms of initial parasite detection (27). This minireview will focus on recent advancements in tuft cell biology as well as examining the potential for other epithelial chemosensory responses to helminths themselves and the microbial dysbiosis infection induces.

## Current Tuft Cell Advancements

Single-cell analysis of the intestinal epithelium now suggests that tuft cells are a heterogenous population, with two proposed distinct subsets—tuft-1 and tuft-2—with differing cytokine profiles (28, 29). In response to undefined helminth antigens, small intestinal tuft cells produce TSLP as well as IL-25, which are crucial for the initiation of the anthelmintic mucosal response (28, 30). Only tuft-2 cells produce TSLP, although a functional role for TSLP from tuft cells has yet to be demonstrated (28). Schneider et al. (31) showed that genetic deletion of tumor necrosis factor alpha-induced protein 3 (*Tnfaip3*), a negative regulator of IL-25 signaling in ILC2, caused tuft cell and goblet cell hyperplasia, as well as small intestinal lengthening. Tissue remodeling that mimics the histological features of a helminth infection (31), reinforcing the importance of IL-25 signaling in type 2 immunity.

The initial identification of the potential taste receptor signaling pathways involved in parasite recognition at the epithelial barrier by Howitt et al. (27) has since been expanded upon. Luo et al. (32) further elucidated the *Trpm5*-dependent sensory pathway by showing that stimulation of intestinal organoids using larvae and antigens of the small intestinal helminth *Trichinella spiralis* stimulates increased intracellular calcium levels, resulting in tuft cell depolarization. They further observed that IL-13 administration promotes tuft cell hyperplasia as well as upregulation of genes including the *Tas2r* family of bitter taste G-protein coupled receptors (GPCRs) and the succinate receptor *Sucnr1*, indicating an adaptive ability of these chemosensory cells during *T. spiralis* infection. The importance of *Tas2r* in tuft cell recognition of helminths was demonstrated when pretreatment of small intestinal villi with allyl isothiocyanate, an inhibitor of bitter taste receptors, abolished *T. spiralis-*induced tuft cell-derived IL-25. Conversely, increased tuft cell production of IL-25 was seen after the administration of salicin, a *Tas2r* agonist (32). Two groups have also demonstrated the importance of *Sucnr1* with Lei et al. (33) demonstrating that tuft cells are the sole epithelial expressers of this receptor in the small intestine. They, in parallel with Schneider et al. (31) demonstrated that dietary succinate increases small intestinal tuft cell secretion of IL-25 and promotes hyperplasia. Interestingly, examination of *Sucnr1* null mice demonstrated a prevention of succinate-induced tuft and goblet cell hyperplasia (33). Nadjsombati et al. (34) demonstrated that succinate metabolites are produced by the small intestinal helminth *Nippostrongylus brasiliensis in vitro*; yet, immunity against *N. brasiliensis* is not abrogated in *Sucnr1*^−/−^ mice, suggesting no requirement or at least redundancy in this potential helminth recognition pathway.

Schneider et al. (31) also reported that small intestinal tuft cells on *Tritrichomonas*-colonized mice highly express not only *Sucnr1* but also GPCRs for short-chain fatty acids *Ffar3*. This result is also corroborated by other groups, who reported that *Ffar3* is highly expressed by intestinal tuft-2 cells, but not intestinal tuft-1 cells or tracheal tuft cells (28, 34). Although the discovery of *Ffar3* expression on small intestinal tuft cells is an interesting find, little is known at the moment on how the receptor impacts anthelmintic immunity. Interestingly, in a murine model of allergic airway inflammation, *Ffar3* knockout abrogates *Heligmosomoides polygyrus*-induced alleviation of airway inflammation but did not affect worm burden in the small intestinal niche of this helminth (35). Murine small and large intestinal tuft cells also express choline acetyltransferase, which catalyzes the production of acetylcholine (36). Although the close interaction between airway tuft cells and cholinergic neurons has been previously demonstrated (37), with recent demonstrations of tuft cell acetylcholine driving ciliary beating in a Trpm5-dependent fashion (38), their role in cholinergic neuron signaling in the intestine is less clear. There is evidence that during genetic and antagonist muscarinic receptor blockade, small intestinal tuft cells arise with an enteroendocrine-like phenotype to sustain the murine intestinal epithelial cholinergic niche (39). Moreover, as acetylcholine receptors are also expressed on diverse cell types, including goblet cells (where acetylcholine promotes mucus secretion), dendritic cells, macrophages, as well as B and T cells (40, 41), there is the unexplored possibility that tuft cells may also play a larger role in anthelmintic immunity *via* their production of acetylcholine.

Recent findings have also begun to elucidate the initial on switch of the tuft cell/ILC2 feed-forward loop, which, given the production of IL-25 in the naive state (24), was likely to be another messenger or danger signal. Tuft cells had previously been shown to produce leukotriene C4 (13), but McGinty et al. (42) have demonstrated that following small intestinal helminth infection, tuft cells secrete leukotriene C4, in an *Alox5*-dependent manner, that could signal to surrounding ILC2s *via* their expression of leukotriene receptors CYSLTR1 and 2 (Figure 1). Given that several immune cells can produce leukotrienes and the long-lived nature of tuft cells (43), bone marrow transfer experiments were superseded by targeted cell-specific null models demonstrating that it was indeed tuft cell-derived leukotriene that was key in driving ILC2 expansion early during small intestinal *N*. *brasiliensis* infection (42). However, the precise chemoreceptor or the helminth products they detect remain unknown. Parallel studies by Ualiyeva et al. (44) have also demonstrated that tuft cells located in the lung can release leukotrienes in response to aeroallergens *via* the P2Y2 receptor, indicating systemic potential for helminth detection. Interestingly, although tuft cells also produce IL-25 in response to protist-derived succinate *via* SUCNR1, McGinty et al. (42) demonstrated that stimulation of tuft cells with succinate, although driving IL-25, resulted in no leukotriene production but importantly no defect in ILC2-driven responses. Furthermore, TAS1R3 also expressed on tuft cells responds to *Tritrichomonas muris* and succinate, but not to a helminth infection (45), indicating an ability of tuft cells to selectively respond to different parasites.

This variety and flexibility of the cellular secretome of tuft cells further mirror the responses of enteroendocrine cells (EECs), key chemosensory cells of nutrient detection, in being able to orchestrate an array of digestive requirements to the numerous nutrients detected. Therefore, it is likely that these pathways have been utilized by the innate immune system in evolution to allow the “tasting” of parasites and allow an equally diverse response in immunity as digestion. Furthermore, EECs demonstrate heterogeneity spatially to respond to the nutrients in the likely locations they would appear (46–49); so it is likely that chemosensory cells detecting and responding to parasites would also differentiate in a spatiotemporal fashion to specific parasite niches along the intestinal tract and beyond, as indicated in spatial studies of tuft cells (28, 29, 36, 50). Tuft cells also possess cytospinules which project into the nuclei of neighboring cells, providing them with a unique ability to communicate cellular cargo to the surrounding epithelium (51). Moreover, tuft cells are often associated with EECs (52), while both cell types can act as reserve stem cell niche in the small intestine upon Paneth cell ablation (53), indicating potential overlap and collective function (Figure 1).

## Enteroendocrine Cells—Key Chemosensory Cells of the Epithelium

EECs are specialized trans-epithelial signal transduction conduits which respond to luminal nutrients by secreting peptide hormones to control gastrointestinal enzyme secretion, motility, and appetite regulation (54, 55). Despite constituting only 1% of the total epithelium, these cells span from the entire length of the gastrointestinal tract and collectively form the largest endocrine system of the body (56). Peptide-secreting intestinal epithelial cells described as having a high degree of amine precursor uptake were reported as early as the 1960s (57). Initially thought to arise from neural crest cells due to their production of neuropeptides such as serotonin, lineage tracing on avian embryos proved that these cells do not arise from the ectoderm (58, 59). Like other intestinal epithelial cells, EECs originate from *Lgr5*^+^ intestinal stem cells within the intestinal crypt, integrating Wnt, Notch, and mitogen-activated protein kinase-dependent signaling (60), and require the expression of the secretory cell lineage transcription factor atonal bHLH transcription factor 1 (61–65), finally forming EECs *via* the expression of the transcription factors neurogenin3 and neurogenic differentiation 1 (NeuroD1) (64, 66, 67). Neurogenin3^+^ EEC progenitor cells will further differentiate to give rise to multiple mature EEC types, traditionally identified with a one-cell one-peptide dogma. This historic classification included glucagon-like-peptide-1 (GLP-1)-producing L-cells, cholecystokinin (CCK)-producing I-cells, gastrin-producing G-cells, gastric inhibitory peptide-producing K-cells, somatostatin-secreting D-cells, secretin-producing S-cells, and serotonin-producing enterochromaffin cells. However, it is now known that there is considerable secretome overlap and plasticity between the different EEC lineages. Using transgenic reporter mice, multiple groups have shown that CCK, GLP-1, and secretin are coexpressed by a large subset of EECs (68, 69). A recent single-cell transcriptional analysis using Neurog3 reporter mice showed that hormonal co-secretion differs by cell lineage, with a large proportion of EECs secreting multiple hormones (70). Furthermore, EECs also show hormonal plasticity in response to various extracellular cues, such as the upregulation of secretin production in response to bone morphogenic protein as well as their physical location within the crypt/villi dictating their secretome (70, 71).

Although still incompletely understood, recent evidence has shown that EECs have a huge potential to interact with the immune system, with a strong potential for playing a role in the chemosensory sensing of helminths and orchestrating immunity (56). Indeed, helminth infections in particular can drive hyperplasia of EECs in a variety of animal species, often thought to be the driving force to alterations in feeding that accompany a helminth infection in the upper small intestine (72–77). These alterations in EEC hyperplasia, like tuft cells, are also driven by type 2 cytokines in both small intestinal (*T*. *spiralis*) and large intestinal (*Trichuris muris*) helminth infections (78–81), with the EEC peptide CCK shown to influence the resulting immune response *via* driving weight loss in a feed-forward loop (81). Moreover, EECs can secrete peptide hormones as well as cytokines in response to pathogen-associated molecules (82), and given that intestinal immune cells potentially express peptide hormone receptors (83–85), there is the intriguing possibility that EECs are critical and novel modulators of barrier immunity to helminths (Figure 1).

Interestingly, EECs are the chief epithelial expressers of the receptors that sense bacterial metabolites, such as Ffar3/2 (86, 87), and therefore have the unique ability to relay dysbiosis into physiological adaptation (88). It is now well-established that microbial dysbiosis occurs during an intestinal helminth infection (89, 90), and these changes are transient following helminth expulsion (91, 92), meaning microbial alterations may provide a clear signal to the epithelium of a helminth infection. Indeed, the microbiota is a well-established essential signal for repair during intestinal inflammation (93) and antibiotic-induced microbial dysbiosis alters succinate levels altering tuft cell numbers in the absence of a helminth infection (33). Microbial load increases greatly in the cecum and large intestine, but small intestinal dysbiosis does occur during a large intestinal helminth infection (94). Although these microbial changes are not as instantaneous as detecting the helminths themselves, they can occur within days of infection (95). Moreover, helminth-driven dysbiosis may actually strengthen existing innate barrier responses, as during large intestinal *Trichuris suis* infection, the addition of the dietary supplement inulin heightens the microbial changes *T*. *suis* initiates (96), resulting in tuft cell hyperplasia (97).

EECs have a heightened ability to potentially sense helminths and/or the microbial dysbiosis they produce *via* the huge array of chemosensory apparatus they possess. Classically, the peptide hormones secreted by EECs signal to the brain in a paracrine fashion *via* local vagal afferents to mediate digestion and satiety. Recently, Bohórquez et al. (98) demonstrated that CCK-expressing EECs possess basal axon-like cytoplasmic processes, termed neuropods, which transpose nutritional and microbial intestinal signals directly to the brain (99). Neuropods are rich in mitochondria, dense secretory vesicles, presynaptic proteins, and neurofilaments and lie in close contact to enteric glia (100). Neuropods are present in both ileal and colonic EECs (98) and have the capacity to respond to and transmit glucose stimuli to vagal neurons in milliseconds (99). The EEC neuropod therefore has the exciting potential to communicate intestinal chemosensory information directly to the brain and, given the novel neurological control of ILCs (101), presents an exciting immunological addition to the gut–brain axis.

## Discussion

Given that RNA-seq analysis of bitter taste receptor-expressing cells in multiple barrier tissues is strongly linked to innate immune transcripts (102), it is clear that we are only at the beginning of fully elucidating the complex interactions of chemosensory, immune, and neuronal cellular interactions during infection. It still remains imperative to define the helminth products that initiate these epithelial cascades which drive immunity. Although tuft cells are reported to respond almost instantly to a helminth infection (42), it remains a possibility that helminth-derived microbial alterations could be a potential slower innate trigger, particularly in the large intestine where reports of helminth-induced tuft cell alterations have so far been absent. Alternatively, chemosensing may fall to EECs in the large intestinal niche and tuft cells may even act in concert with EECs utilizing microspinule communication to harness neighboring EECs neuropod signaling to help drive the systemic immunity often seen during a helminth infection. In summary, the initial fascinating epithelial chemosensory discoveries discussed above could simply be a taste of things to come.

## Author Contributions

JW contributed to the conceptualization. AF, KW, JT, and JW contributed to writing the original draft. JW contributed to the writing, review, and editing. All authors contributed to the article and approved the submitted version.

## Conflict of Interest

The authors declare that the research was conducted in the absence of any commercial or financial relationships that could be construed as a potential conflict of interest.
